# Exploring the Effect of Whole-Genome Duplication on Salmonid LincRNA Repertoire

**DOI:** 10.3390/ijms27177861

**Published:** 2026-09-02

**Authors:** Isabel García-Pérez, Daniel Garcia de la serrana

**Affiliations:** Department of Cell Biology, Physiology and Immunology, Faculty of Biology, University of Barcelona, 08028 Barcelona, Spain; isabelgarcia@ub.edu

**Keywords:** long intergenic non-coding RNA, ohnologues, salmonids, secondary structure

## Abstract

Long intergenic non-coding RNAs (lincRNAs) are key epigenetic regulators of genome function, yet their evolutionary dynamics following whole-genome duplication (WGD) events remain poorly understood. Salmonids, which underwent a lineage-specific autotetraploidization (salmonid-specific WGD, ~88–100 million years ago), provide an excellent model to investigate the retention, divergence, and functional potential of recently duplicated non-coding elements. LincRNA repertoires were compared across five genome-annotated salmonids (*Oncorhynchus tshawytscha*, *O. kisutch*, *O. mykiss*, *Salmo salar*, and *S. trutta*) and their closest non-duplicated relative, northern pike (*Esox lucius*). LincRNAs represented ~5–7% of annotated genes in all salmonids except *S. salar* (18%). Sequence conservation was low relative to coding genes, with only 11–68 highly similar (e-value < 1 × 10^−30^; similarity > 70% and alignments > 100 nucleotides) putative orthologues shared between salmonids and northern pike, and 161–338 among salmonids alone. Synteny conservation was modest in lincRNAs, with lower conservation in putative orthologues (8–16%) compared to putative ohnologues (8–33%). Secondary structure conservation was associated with sequence similarity (*ρ* = −0.45; *p* = 2.2 × 10^−16^), and the association was stronger among WGD ohnologues than orthologues. In *S. salar* and *O. mykiss*, lincRNA putative ohnologues showed weaker expression correlations than coding genes, suggesting widespread regulatory divergence, possibly through neo- and subfunctionalisation. Conserved salmonid lincRNAs showed enriched predicted interactions with miRNAs involved in tumour suppression, brain, bone, and muscle development (e.g., *miR-455*, *miR-365*, *miR124*, *miR-133a*, *miR-140*, and *miR-9*), a finding supported by limited transcriptomic data. Although salmonid WGD expanded lincRNA repertoires, lincRNAs have undergone rapid sequence and transcriptional divergence, with limited conservation across species based on sequence similarity, chromosomal position, synteny, and secondary structure. A subset of conserved lincRNAs retains structural features and regulatory signatures consistent with roles as miRNA sponges in brain, skeletal, and muscle development and tumour suppression, potentially acting within conserved regulatory networks. These findings provide new insights into lincRNA evolution following genome duplication and highlight the need for experimental validation of their regulatory functions.

## 1. Background

Long intergenic non-coding RNAs (lincRNAs) represent a class of non-coding transcripts exceeding 200 nucleotides in length, typically originating from genomic regions located between protein-coding genes [1,2,3]. Although they lack protein-coding potential, they serve as sophisticated regulatory hubs, modulating gene expression across diverse transcriptional and post-transcriptional layers [3,4]. These molecules exert their functions through a versatile repertoire of mechanisms, often classified as signals, decoys, scaffolds, and guides, enabling both cis- and trans-acting functionalities [3,4]. For instance, lincRNAs can recruit or sequester chromatin-remodelling complexes, such as the Polycomb Repressive Complex 2 (PRC2), or form RNA–DNA triplexes to regulate transcriptional access to transcriptional machinery. Beyond nuclear functions, they influence cytoplasmic processes by acting as molecular sponges for microRNAs (miRNAs), thereby buffering miRNA availability, or by directly modulating mRNA stability, translation, and splicing kinetics [3,4].

While lincRNA research has gained considerable momentum in development and human disease, our understanding of teleost lincRNAs remains largely confined to the zebrafish (*Danio rerio*) (Hamilton, 1822) model [5,6]. Recent studies, however, have begun to elucidate their importance across teleosts. In the spiny chromis damselfish (*Acanthochromis polyacanthus*) (Bleeker, 1855), for example, lincRNAs have been implicated in the adaptive response to ocean acidification, while studies in species such as rainbow trout (*Oncorhynchus mykiss*) (Walbaum, 1792) and turbot (*Scophthalmus maximus*) (Linnaeus, 1758) highlight their involvement in mediating responses to heat stress and immune challenges [7]. Other studies in salmonids and non-salmonid species have also described how lincRNAs might modulate skeletal muscle growth [8,9].

Despite their regulatory significance, lincRNAs are characterised by rapid primary sequence evolution and generally display lower expression levels, fewer exons, and reduced sequence conservation than protein-coding genes [10,11]. This rapid evolutionary turnover presents significant challenges for the identification of orthologues and the application of traditional “guilt-by-association” functional inference, making comparative analyses across species particularly challenging [12,13]. Notably, while primary sequences diverge quickly, some studies suggest that secondary structural conservation may provide a more reliable basis for assessing evolutionary and functional homology, as illustrated by the identification of the mitochondrial *lncOriL* in both zebrafish and humans [14].

The evolutionary history of these non-coding RNAs (ncRNAs) is further complicated by whole-genome duplication (WGD) events, which have fundamentally shaped the vertebrate genome. Following the two ancestral rounds of WGD (1R and 2R) at the base of the vertebrate lineage, approximately 500–520 Mya (million years ago), the third round (3R) took place approximately 320–350 Mya (TWGD) in the lineage leading to modern teleosts before the emergence of the earliest known teleost fossils [15,16]. After the TWGD, other duplication events took place, including the salmonid-specific WGD (SWGD; 4R) roughly 88–103 Mya [16,17,18]. The fate of the resulting ohnologues (duplicates generated after a WGD event) is typically defined by gene loss via pseudogenization or retention through mechanisms such as subfunctionalisation, neofunctionalisation, or functional redundancy [19,20]. While an estimate of only 12–24% of protein-coding gene duplicates generated by the TWGD (3R) have been retained in extant teleost lineages, retention following the SWGD (4R) is markedly higher, reaching estimates up to 48–55% [21,22,23].

Analogous to coding genes, ncRNAs would also be subject to duplication and rediploidization during a WGD event. Analyses in salmonids have revealed extensive retention of small ncRNA repertoires following SWGD, with many miRNA loci displaying higher retention than even coding genes, likely reflecting the strong selective constraints on maintaining regulatory network stability to buffer potential gene dosage imbalances resulting from WGD [21,24]. However, the specific evolutionary trajectories of lincRNAs following WGD events remain poorly defined. Given their intrinsic propensity for rapid divergence, it remains to be determined whether these ohnologues undergo accelerated pseudogenization or substantial functional divergence compared to their coding counterparts.

In addition to lincRNAs, miRNAs (ncRNAs around 20 nucleotides long) serve as fundamental post-transcriptional regulators, orchestrating complex gene networks, mainly through sequence-specific binding to the 3′ untranslated regions (UTRs) of target mRNAs. Unlike the rapid sequence turnover typical of lincRNAs, mature miRNAs exhibit evolutionary conservation, with many seed sequences remaining highly conserved across vertebrate evolution [25,26]. This stability underscores their role as essential modulators of cellular homeostasis, particularly in buffering gene expression levels within signalling pathways governing development, tissue differentiation, and stress responses [27]. The functional interplay between lincRNAs and miRNAs represents a critical layer of post-transcriptional regulation, characterised by a complex competition for target access. Within this network, lincRNAs frequently function as endogenous competing RNAs or molecular sponges, possessing miRNA-binding sites that enable them to sequester mature miRNAs away from their canonical protein-coding targets [28]. By modulating the local availability of miRNAs, lincRNAs effectively derepress these target mRNAs, thereby fine-tuning the output of signalling cascades. This sponge mechanism is of particular significance in the wake of WGD events; as the salmonid genome navigates the retention and expression of duplicated ohnologues, the expansion of lincRNA loci may provide an additional regulatory buffer that modulates miRNA availability.

In the present study, we investigated the impact of the WGD on highly similar lincRNA (likely originated during the SWGD) abundance and retention across five salmonid genomes (*Salmo salar* [Linnaeus, 1758], *Salmo trutta* [Linnaeus, 1758], *Oncorhynchus mykiss* [Walbaum, 1792], *Oncorhynchus kisutch* [Walbaum, 1792], and *Oncorhynchus tshawytscha* [Walbaum, 1792]), together with the northern pike (*Esox lucius*) [Linnaeus, 1758] as the closest non-duplicated relative. By analysing lincRNA distribution, orthology, local synteny, conservation of miRNA interactions, and transcriptomic data, we evaluated whether duplicated lincRNAs maintain structural and functional coherence or whether they follow a path of rapid divergence and neofunctionalisation, using both bioinformatic and transcriptomic analyses. However, comparable transcriptomic resources were only available for two of the species of the present study (Atlantic salmon and rainbow trout), limiting the scope of this study.

The present study hypothesised that the recent salmonid SWGD increased lincRNA repertoire size and retained duplicated lincRNA pairs that, while undergoing more rapid sequence and transcriptional divergence than protein-coding genes, nevertheless retained a higher degree of conservation. It was further hypothesised that these conserved lincRNAs retained function through preservation of structure and miRNA-mediated regulatory interactions.

## 2. Results

### 2.1. Distribution of lincRNAs in Salmonid Genomes

**Figure 1:**
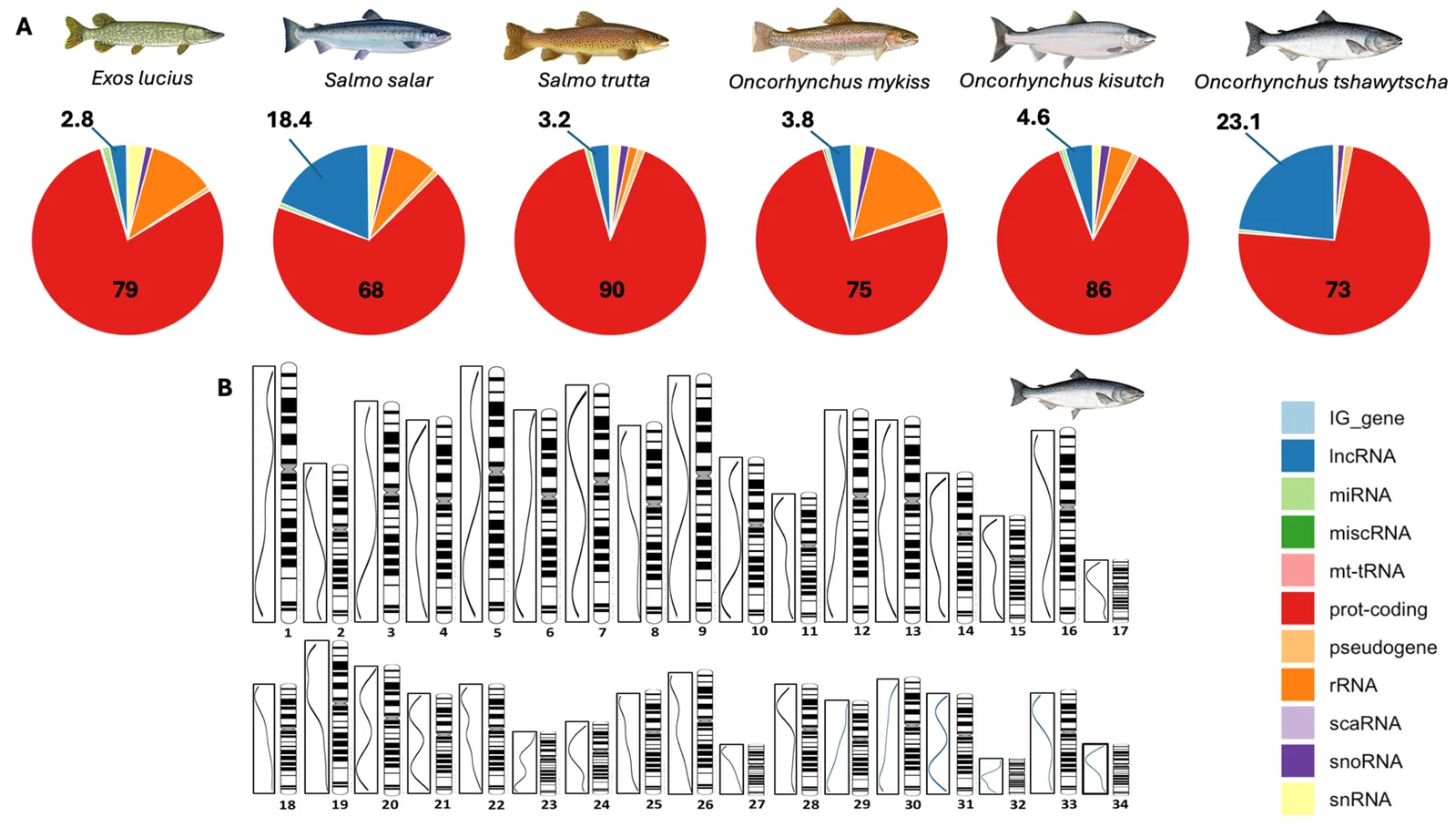
LincRNA distribution in Salmoniformes and the non-SWGD-duplicated outgroup northern pike. (**A**) Relative proportions of annotated gene categories (snRNA, snoRNA, scaRNA, rRNA, pseudogenes, protein-coding genes, mtRNAs, miscRNAs, miRNAs, lincRNAs, and IG genes) in the genomes of five salmonid species and the non-duplicated outgroup northern pike. The percentage of protein-coding genes (red) and lincRNAs (blue) is indicated for each species. (**B**) LincRNA density is represented by the density plots shown within each chromosome across the Atlantic salmon karyotype. Chromosome lengths were scaled according to information available in Ensembl. Chromosome banding is schematic and does not represent actual cytogenetic banding patterns. The illustration is not intended to represent DNA sequence information.

The total number of annotated lincRNAs present in the salmonids and northern pike genomes varied substantially among some species. In salmonid genomes, lincRNAs represented around 5–7% of all annotated genes, almost twice that observed in northern pike (2.9%), their closest non-salmonid relative. Atlantic salmon, however, showed a markedly higher proportion of lincRNAs compared with the other salmonids (18%) (Figure 1A). Atlantic and Chinook salmon also had the highest absolute numbers of lincRNAs (12,700 and 13,650, respectively), exceeding those of the other salmonids (1500–3500) and northern pike (900) (Figure 1A). The significantly larger lincRNA repertoires observed in Atlantic and Chinook salmon may partly be the consequence of genome assembly quality, annotation depth, transcriptomic support and gene-model prediction pipelines among genome assemblies rather than true biological differences; however, further analyses would be necessary to distinguish between these possibilities.

The distribution of lincRNA location across the salmonid karyotype was relatively homogeneous (Figure 1B). However, a relatively higher number of lincRNAs was evident towards many chromosome arm ends and was much lower in centromeric regions (Figure 1B).

**Figure 2:**
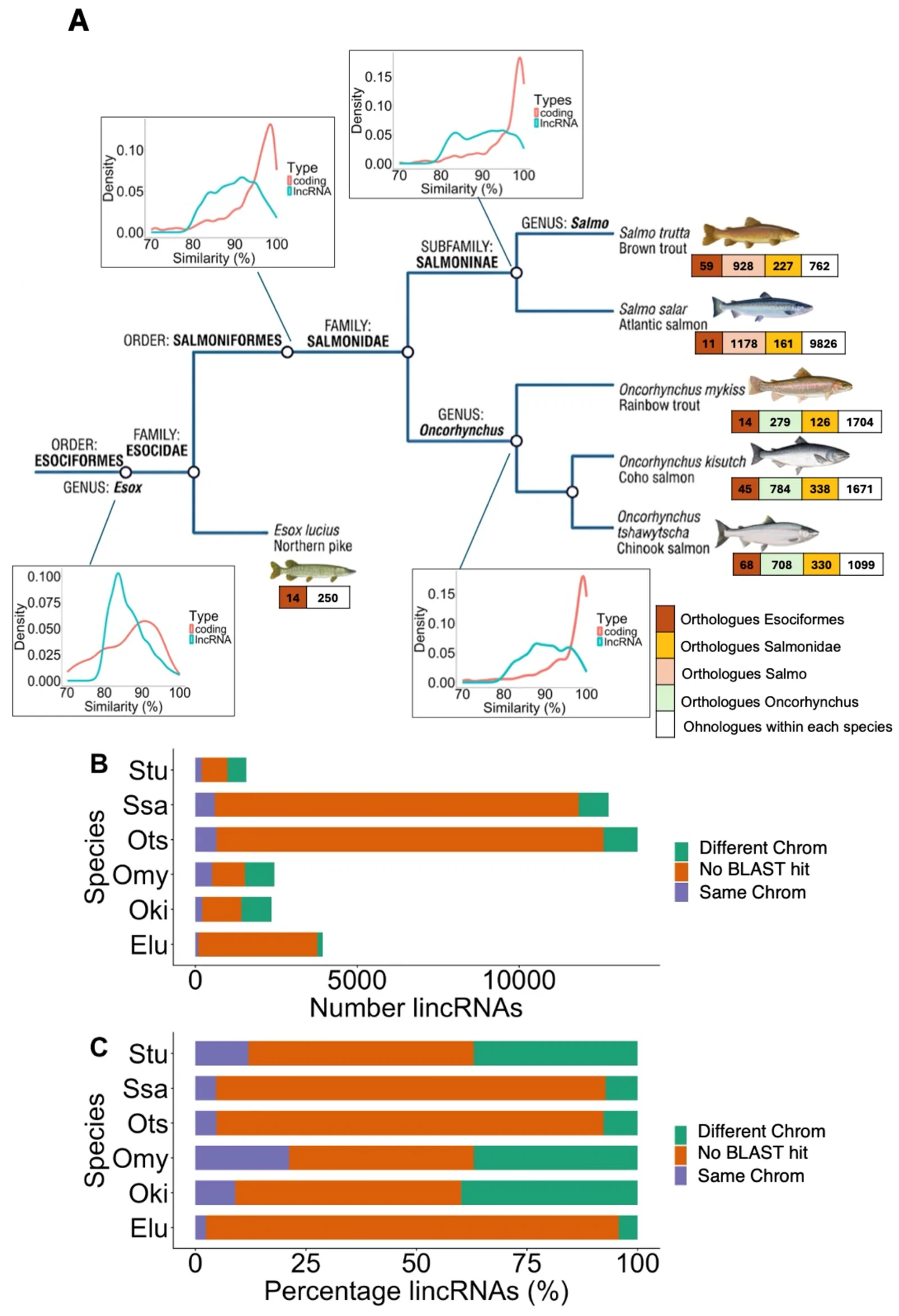
Orthologous relationships and highly similar duplicated lincRNAs in salmonid genomes. (**A**) Schematic non-metric cladogram representing the evolutionary relationships among five Salmoniformes and the non-duplicated outgroup northern pike [18]. Branch lengths do not represent evolutionary time and are shown for clustering purposes only. Orders, families, genera, and subfamilies are indicated. Inset density plots show the distribution of sequence-similarity values for lincRNA orthologues (green) and protein-coding orthologues (red). The position of the graph indicates the species involved in the orthologues’ similarity analyses. Values inside coloured boxes indicate the number of highly similar (e-value < 1 × 10^−30;^ similarity > 70% and over >100 nucleotides alignment) orthologues and ohnologues. (**B**) Total number of non-duplicated lincRNAs (orange), duplicated lincRNAs located on the same chromosome (purple), and duplicated lincRNAs located on different chromosomes (green), for each species analysed: *Ssa* (*Salmo salar*), *Omy* (*Oncorhynchus mykiss*), *Stu* (*Salmo trutta*), *Oki* (*Oncorhynchus kisutch*), *Ots* (*Oncorhynchus tshawytscha*), and *Elu* (*Esox lucius*). (**C**) Percentage of non-duplicated lincRNAs (orange), duplicated lincRNAs located on the same chromosome (purple), and duplicated lincRNAs located on different chromosomes (green), for each species analysed: *Ssa* (*Salmo salar*), *Omy* (*Oncorhynchus mykiss*), *Stu* (*Salmo trutta*), *Oki* (*Oncorhynchus kisutch*), *Ots* (*Oncorhynchus tshawytscha*), and *Elu* (*Esox lucius*).

BLASTn analyses of annotated lincRNAs between salmonids and northern pike, designed to detect highly similar lincRNAs, revealed that the number of potential lincRNA orthologues increased with phylogenetic proximity (Figure 2A) (Supplementary File S1). The number of similar lincRNAs with potential orthologous relationships across salmonids and northern pike ranged from just 11 to 68 depending on the species, whereas the number shared exclusively among salmonids (excluding northern pike) ranged from 161 to 338 (Figure 2A) (Supplementary File S1). Consistent with their evolutionary relationships, putative orthology relationships between lincRNAs were highest within genera: 928–1178 pairs in *Salmo* and 279–784 in *Oncorhynchus* (Figure 2A). Sequence similarity among putative lincRNA orthologues increased with phylogenetic proximity, with the lowest values observed in comparisons involving northern pike. Accordingly, comparisons between more closely related species had a higher proportion of lincRNAs showing >90% identity, but always significantly lower than coding genes (Figure 2A).

Local synteny conservation among putative highly conserved lincRNA orthologues was also investigated. Between 8 and 16% of salmonid lincRNA orthologues showed conserved synteny, comparable to pairwise comparisons with northern pike (5%) (Table 1) (Supplementary File S1).

### 2.2. Putative lincRNAs and Ohnologues

All lincRNAs within each species were subjected to self-BLASTn searches to estimate the number of highly conserved lincRNA duplicates in each genome. The number of highly similar duplicated lincRNAs in northern pike was relatively low (248) compared to salmonids (1497, 1270, 717, 1604, and 1093 in Atlantic salmon, rainbow trout, brown trout, Chinook salmon, and coho salmon, respectively) (Figure 2B; Supplementary File S2). Based on the relative genomic positions of duplicated lincRNAs, two possible duplication mechanisms were initially proposed to simplify downstream analyses (secondary structure conservation): lincRNA pairs located on the same chromosome were initially classified as potential local duplications (LDs), whereas those located on different chromosomes were considered putative ohnologues derived from a WGD (Figure 2B). To independently assess candidates identified through sequence similarity and chromosomal location, conserved syntenic blocks were identified using MCScanX (v1.0.0) and subsequently verified by manual inspection. Between 8.1% and 15.0% of the highly similar duplicated lincRNAs were located within conserved syntenic blocks, depending on species (with a global synteny conservation of 8–33% when all lincRNAs, and not only highly similar ones, were considered) (Table 2). Although this proportion represents only a subset of all putative ohnologues, the presence of conserved genomic context provides additional evidence supporting their potential origin from the salmonid-specific whole-genome duplication (Supplementary File S2).

Future analyses should further validate these putative lincRNA ohnologues using synonymous divergence estimates (Ks) and phylogenetic analyses.

In all salmonid species, the total number of highly similar lincRNA ohnologues exceeded the number of potential LD-derived duplicates, whereas in northern pike the numbers in these were quite similar (Figure 2B). The proportion of putative lincRNA ohnologues relative to the total lincRNA repertoire varied among species, ranging from less than 15% in Atlantic salmon and Chinook salmon to approximately 30–40% in rainbow trout, brown trout, and coho salmon (Figure 2C).

### 2.3. Conservation of Secondary Structure in Duplicated lincRNAs

Secondary structures of predicted orthologous and ohnologous lincRNAs were compared to assess structural conservation. Only lincRNA pairs with ≥50% sequence alignment were included in the secondary-structure modelling. This criterion was chosen based on preliminary tests performed by our group using non-salmonid datasets, which suggested that lower alignment coverage generally failed to produce reliable structural models.

**Figure 3:**
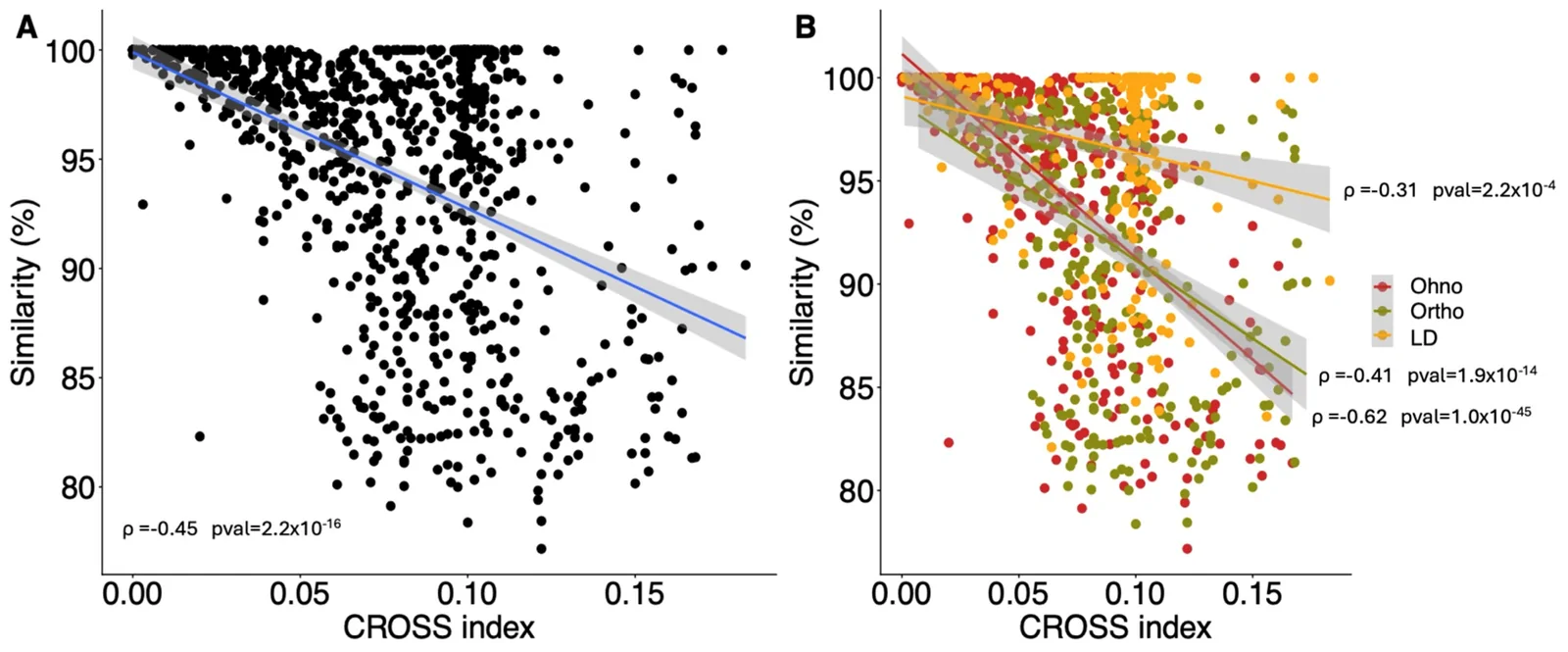
Correlation between sequence similarity and secondary structure conservation in lincRNA pairs. (**A**) Relationship between sequence similarity (%) and the secondary-structure conservation index (CROSS score) for highly similar lincRNA pairs (*n* = 970). The blue line represents the fitted regression, and the shaded region indicates the 95% confidence interval. Correlation line (blue) between the data and confidence interval (blue shade) (*n* = 970). Pearson correlation coefficient and *p*-value are indicated. (**B**) Relationship between sequence similarity (%) and the CROSS structural-conservation index for lincRNA orthologues (green; *n* = 306), putative local duplicates (LDs; yellow; *n* = 237), and putative ohnologues (red; *n* = 427). Regression lines and their corresponding confidence intervals are shown using the same colour scheme. Pearson correlation coefficient (*ρ*) and associated *p*-values for each group are indicated adjacent to the corresponding regression line.

**Figure 4:**
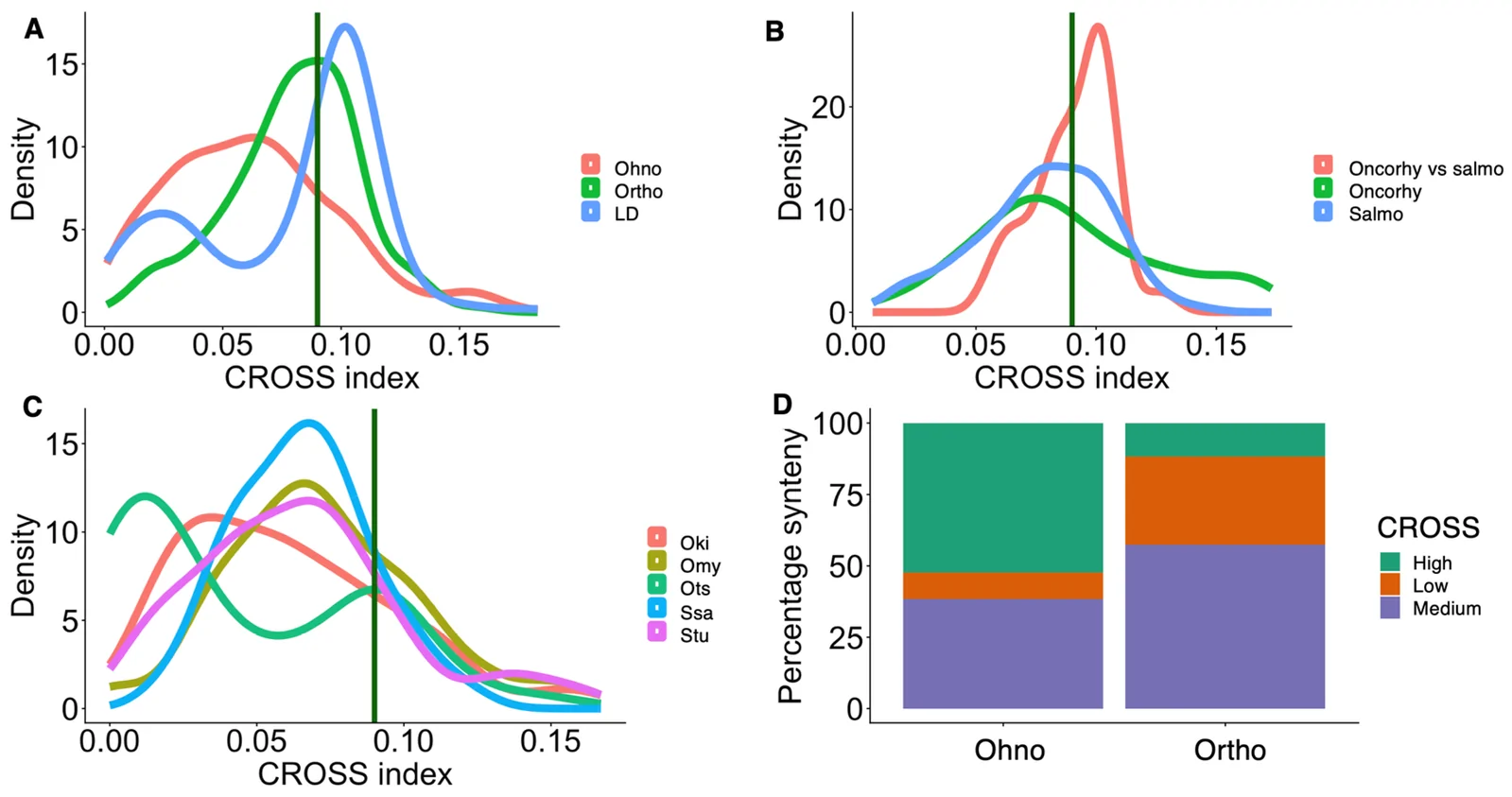
Conservation of secondary structure in lincRNA orthologues and ohnologues. (**A**) Density distribution of the secondary structure conservation (CROSS index values) among highly similar (e-value < 1 × 10^−30^, similarity > 70% and >100 nucleotides alignment) lincRNA orthologues (Ortho; green line) (*n* = 306), putative ohnologues (Ohno; red line) (*n* = 427) and putative local duplicates (LD; blue line) (*n* = 237). The vertical line indicates the CROSS index of 0.09, below which secondary structure was considered conserved. (**B**) Density distribution of secondary structure conservation (CROSS index values) among lincRNA orthologue pairs within the genera *Salmo* (blue line) (*n* = 84), *Oncorhynchus* (Oncorhy; green line) (*n* = 191) or comparing orthologues between *Salmo* and *Oncorhynchus* genera (red line) (*n* = 31) orthologues. The vertical green line indicates the CROSS index of 0.09, below which a secondary structure is conserved. (**C**) Density distribution of the secondary structure conservation (CROSS index values) between highly similar lincRNAs duplicate pairs within each salmonid species: *Salmo salar* (*Ssa*; blue line) (*n* = 39), *Salmo trutta* (*Stu*; pink line) (*n* = 68), *Oncorhynchus mykiss* (*Omy*; brown line) (*n* = 158), *Oncorhynchus kisutch* (*Oki*; red line) (*n* = 82), and *Oncorhynchus tshawytscha* (*Ots*; green line) (*n* = 80). The vertical line indicates the CROSS index of 0.09, below which a secondary structure was considered conserved. (**D**) Percentage of highly similar lincRNA putative ohnologue pairs within each species (ohnologues; Ohno) and orthologues (Ortho) with conserved synteny categorised according to their degree of conserved secondary structure as low (CROSS index 0.05–0.09; orange), medium (CROSS index 0.03–0.05; purple), and high (CROSS index < 0.03; green). Density distribution and chi-square statistics can be found in Supplementary File S3.

This approach yielded 427 putative ohnologue pairs, 237 putative LD pairs, and 306 putative orthologue pairs suitable for secondary structure conservation analysis between pairs (Figure 3 and Figure 4). Structures were classified as highly conserved (CROSSalign index 0–0.03), moderately to poorly conserved (0.03–0.09), or not conserved (>0.09) to facilitate interpretation of the results.

Secondary structure conservation among lincRNA pairs (*n* = 970) was correlated with sequence similarity in the aligned region (average *ρ* = −0.45; *p* = 2.2 × 10^−16^) (Figure 3A) and percentage of sequence overlap (*ρ* = −0.23; *p* = 4.1 × 10^−13^), but not with other features such as sequence length (*ρ* = −0.05; *p* = 0.09) or GC content (ρ = 0.09; *p* = 0.06) (Supplementary File S3). The dispersion of data points across decreasing levels of sequence similarity suggested that lower sequence similarity reduced the likelihood of secondary structure conservation (Figure 3A,B), with structural conservation declining sharply when similarity fell below 80% (Figure 3A,B) (Supplementary File S3), further supporting the choice of a >70% similarity threshold selected for analyses of secondary structure conservation between lincRNA pairs.

Due to the association between sequence similarity and structural conservation, ohnologues, which were on average more similar, exhibited a higher trend to conserve the secondary structure than orthologues (Figure 4A) and were significantly different from putative orthologues (K-test *p* = 6.4 × 10^−10^) and LD (K-test *p* = 2.2 × 10^−16^). Notably, LD lincRNAs showed a binomial distribution regarding the conservation of the secondary structure, one with low secondary structure conservation and another with high secondary structure conservation (Figure 4A). Among orthologues, structural conservation was similar between *Salmo* and *Oncorhynchus* (K-test *p* = 0.022) genera but higher within species of the same genus (Figure 4B) (Supplementary File S3). CROSSalign index distributions among putative ohnologues showed a general tendency for lincRNA pairs to have secondary structure index around 0.05 (Figure 4C). The distribution of secondary structure conservation was quite similar between Atlantic salmon, brown trout, and rainbow trout, while coho and Chinook salmon showed slightly different distributions (K-test *p* < 0.001) (Figure 4C) (Supplementary File S3).

Notably, approximately 50% of putative ohnologue pairs exhibiting highly conserved secondary structure also retained conserved local synteny (Figure 4D; *p* = 6.1 × 10^−4^).

### 2.4. Transcriptional Correlation Between Duplicated lincRNAs

Conservation of expression profiles may provide insights into the molecular mechanisms underlying the retention of putative duplicated lincRNAs. To this end, we used the extended transcriptomic data for Atlantic salmon and rainbow trout generated during the EU AQUA-FAANG project to examine transcription patterns between potential ohnologues and orthologues for rainbow trout and Atlantic salmon [29]. As expected, the majority of lincRNA potential duplicates in both species exhibited very low transcription levels (<1 TPM) (62% in rainbow trout; 50% in Atlantic salmon). A total of 1207 Atlantic salmon lincRNA pairs and 1169 rainbow trout lincRNA pairs satisfied the expression threshold criteria and were included in correlation analyses.

**Figure 5:**
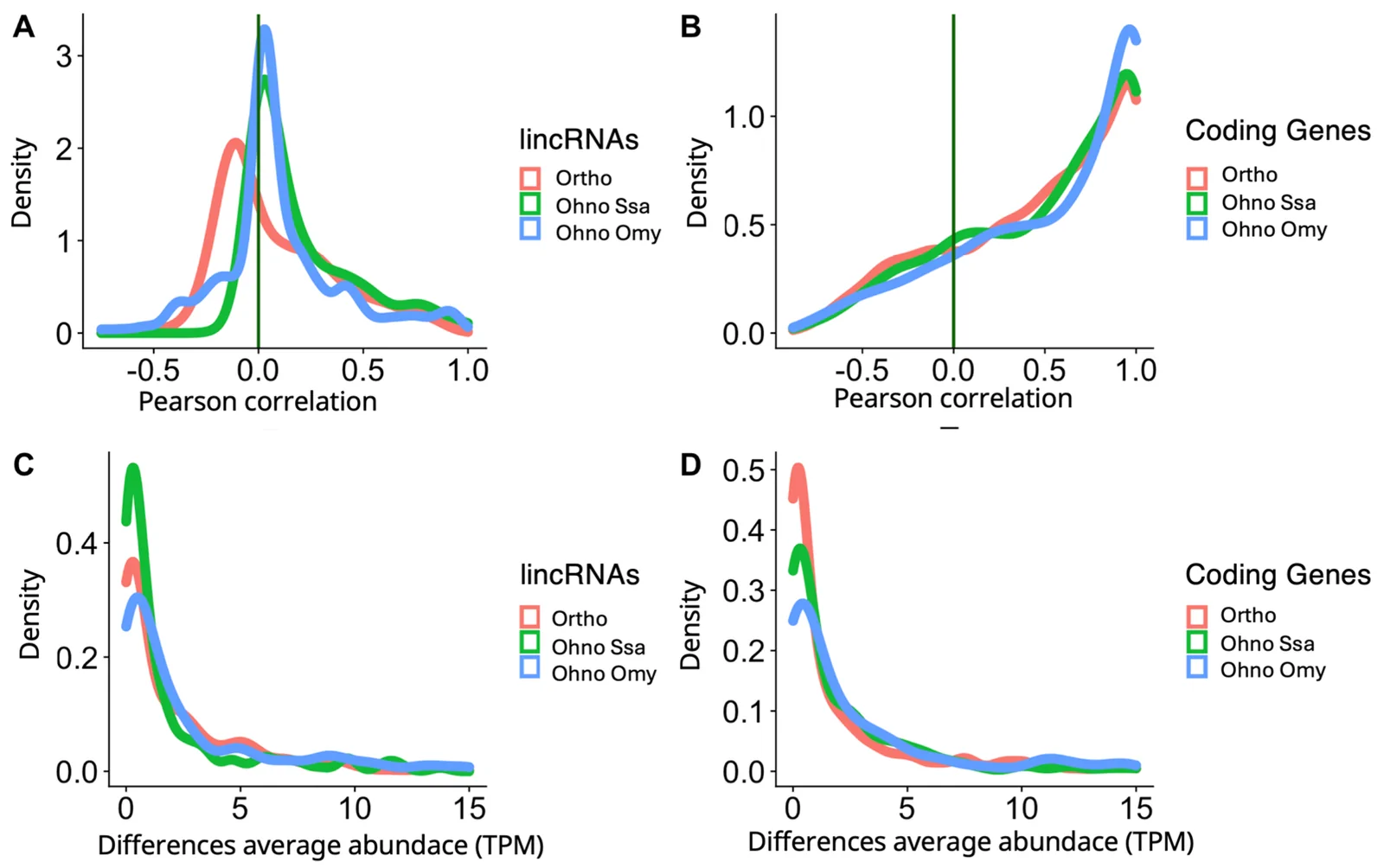
Expression correlations and transcriptional differences among lincRNA and coding gene orthologues and ohnologues in Atlantic salmon and rainbow trout. (**A**,**B**) Density distributions of the Pearson correlation coefficients for highly similar (e-value < 1 × 10^−30;^ similarity > 70% and >100 nucleotide alignment) ohnologue pairs in Atlantic salmon (green; Ohno *Ssa*) and rainbow trout (blue; Ohno *Omy*), and orthologues (red; Ortho) lincRNAs (**A**) and coding genes (**B**). The vertical green line indicates the position of the Pearson coefficient = 0. (**C**,**D**) Density distribution of the differences in transcription (TPM) coefficients for highly similar (e-value < 1 × 10^−30^; similarity > 70% and >100 nucleotide alignment) ohnologue pairs in Atlantic salmon (green; Ohno *Ssa*) and rainbow trout (blue; Ohno Omy), and orthologues (red; Ortho) lincRNAs (**C**) and coding genes (**D**).

Atlantic salmon and rainbow trout similar lincRNA ohnologues exhibited predominantly weak correlations (between −0.1 and 0.1; Figure 5A) compared to orthologous lincRNA pairs (K-test *p* = 2.2 × 10^−16^ and 2.2 × 10^−11^ for Atlantic salmon and rainbow trout respectively), with strong negative correlations (*ρ* < −0.5) relatively infrequent, whereas positive correlations were more common (Figure 5A) (Supplementary File S3). When Atlantic salmon and rainbow trout putative lincRNA orthologues were compared, a similar pattern was found, with a majority of weak correlations (between 0.1 and −0.1) and a slightly larger proportion of negative correlations (*ρ* < 0), but with rainbow trout showing a higher proportion of negative correlations compared to Atlantic salmon (K-test *p* = 8.2 × 10^−5^) (Figure 5A) (Supplementary File S3). In contrast, ohnologues and orthologues from coding gene ohnologues and orthologues from Atlantic salmon and rainbow trout tended to show positive correlations (ρ > 0.5; Figure 5B) (Supplementary Files S3 and S4). Differences in average transcription levels (TPM) were also assessed in embryonic samples, where many lincRNAs were expressed. Most ohnologues, regardless of whether they were lincRNAs or protein-coding genes, showed small differences at the transcription level between pairs (<5 TPM), whereas larger differences (>100 TPM) were less common; differences over 1000 TPM were extremely rare (Figure 5C) (Supplementary Files S3 and S4). Orthologues between the two species also showed the same trend, but interestingly, the number of lincRNA pairs with differences < 5 TPM was higher than in CDS (Figure 5D; Supplementary Files S3 and S4). Notably, lincRNA pairs from Atlantic salmon showed a higher proportion of pairs with very small differences in expression compared to rainbow trout (K-test *p* = 5.1 × 10^−6^) (Supplementary File S3).

For visualisation purposes, density plots were truncated to 15 TPM. Density distribution statistics and complete plots are provided in Supplementary File S4.

### 2.5. Conservation of miRNA Interactions

The possible function of lincRNAs as miRNA sponges was also studied using potentially conserved lincRNAs across the five salmonid species (identified by a BLAST-based approach and excluding northern pike) against all annotated miRNAs available in Ensembl (ensembl.org; accessed on 15 July 2025) (Supplementary File S5). The interaction analysis showed that conserved salmonid lincRNAs showed the strongest predicted interactions with 18 miRNAs: *miR-455*, *miR-455b*, *miR-365a*, *miR-365-1*, *miR-153a*, *miR-153-2*, *miR-124-2*, *miR-124-3*, *miR-140*, *let-7*, *miR-9-3*, *miR-2*, *miR-15a-1*, *miR-1306*, *miR-192b*, *miR-30b*, *miR-133a*, and *miR-98* (Table 3; Supplementary File S5). While those 18 miRNAs had the strongest interactions, the results also showed a predominance of strong interactions towards miRNAs linked to bone (*miR-455*, *miR-140*, and *miR-365*), neurogenesis (*miR-9*, *miR-124*, and *miR-153*), and muscle development (*miR-133a* and *miR-22*) and, to a lesser extent, with miRNAs associated with tumour suppression (*miR-15a*, *miR-30b*, *miR-192*, *miR-98*, and *let-7*) (Supplementary File S5). Similarly, Atlantic salmon and rainbow trout orthologues with correlations over 0.80 also showed strong interactions for the same set of miRNAs, especially *miR-455*, *miR-365a*, *miR-124*, and *miR-133a*. Similarly, when highly similar ohnologue pairs within each species were analysed for their interactions with miRNAs, they showed the strongest interactions with the same set of miRNAs (Supplementary File S6).

Because a great number of strong interactions were found in lincRNA orthologues and ohnologues with bone- and brain-related miRNAs, the sequences of a total of 46 key genes involved in bone and brain regulation and development were also downloaded for all 5 salmonid species (Supplementary File S7), and their interactions with miRNAs and lincRNAs were investigated. Strong interactions (MFE threshold < −50 kcal/mol) were detected for most genes analysed, although Atlantic salmon and rainbow trout showed a larger number of genes without detectable interactions (Supplementary File S7), against *miR-455*, *miR-153*, *miR-365a*, and also *miR-133a* (Supplementary File S7). These included key regulators of bone and brain development such as *alpl*, *bdnf*, *col1a2*, *hdac4*, *neurod1*, *spp1*, *sav1*, *sost*, or *sparc* (Table 4; Supplementary File S7), whereas no interactions were detected for other key genes such as *runx2* or *fbn1*.

**Figure 6:**
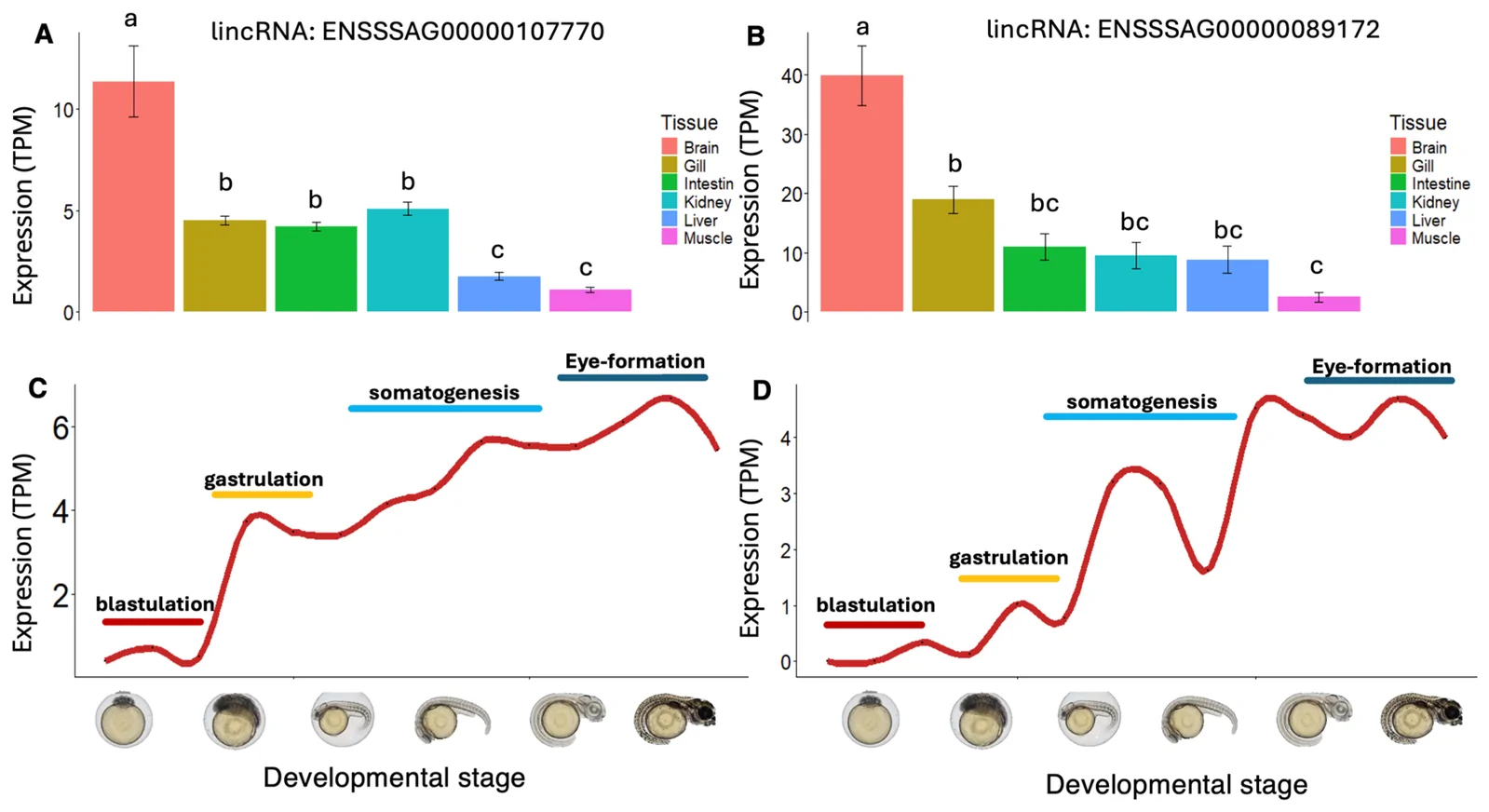
Expression profiles of conserved lincRNAs in Atlantic salmon tissues and embryonic development. (**A**,**B**) Transcriptional abundance of *Salmo salar* (*Ssa*) lincRNAs ENSSSAG00000107770 and ENSSSAG00000089172 in adult Atlantic salmon brain, gill, intestine, kidney, liver, and skeletal muscle tissue. Data are expressed as average values of normalised RNAseq reads (TPM) and standard deviation (*n* = 12). Different letters indicate statistically significant differences among tissues (*p* < 0.05). (**C**,**D**) Transcriptional abundance of lincRNAs ENSSSAG00000107770 and ENSSSAG00000089172 during Atlantic salmon embryonic development: early blastula, mid-blastula, late blastula, early gastrulation, mid-gastrulation, late gastrulation, early somatogenesis, early–mid-somatogenesis, mid-somatogenesis, mid–late somatogenesis, late somatogenesis, early eye formation, and late eye formation. Data represent individual values (*n* = 3) of normalised RNAseq reads (TPM).

The transcription of lincRNAs with potential sponge function was further investigated in the AQUA-FAANG transcriptomic data during embryonic development and in adult tissues from Atlantic salmon and rainbow trout. A total of 32 and 17 lincRNAs were found to be more highly expressed in Atlantic salmon and rainbow trout brain, respectively, compared to other tissues, whereas only 10 and 3 lincRNAs were found to be more highly expressed during early and mid-somatogenesis (formation of the brain) and early and late eye formation (where formation of the bone happens) of the embryo (e.g., Figure 6).

Similarly, the possible correlations between conserved lincRNAs in Atlantic salmon and rainbow trout and key bone and brain genes, using AQUA-FAANG transcription data, were studied. The results showed that 51 and 7 lincRNAs in Atlantic salmon and rainbow trout, respectively, were strongly correlated (*ρ* > 0.80) with 60 and 41 transcripts of key brain and bone genes (Supplementary File S7). Interestingly, no strong negative correlations (*ρ* < −0.80) were found between conserved lincRNAs and brain and bone genes.

The direct interactions between lincRNAs conserved across salmonids and key bone and brain genes were also evaluated. Interestingly, the number of possible interactions between lincRNAs and mRNAs varied greatly between species, with Chinook salmon showing the lowest number of predicted interactions (just 2), whereas brown trout showed 110 predicted interactions (Supplementary File S7). Only two genes showed conserved lincRNA–mRNA interactions across species: col1a2 (where interactions were found in all but Chinook salmon, with an average interaction strength of −0.12 kcal × mol^−1^ × nt^−1^) and *spp1* (present in all species with a predicted average interaction strength of −0.22 kcal × mol^−1^ × nt^−1^) (Table 4; Supplementary File S7).

## 3. Discussion

This study provides a preliminary comparative analysis of lincRNA duplication across salmonids. By integrating sequence similarity, genomic context, structural conservation, interactions, and transcriptional data, our results highlight both the contribution of genome duplication to lincRNA repertoires and the divergence–conservation dynamics of these ncRNAs. Substantial variation was observed in the number of identified lincRNAs across species, with markedly higher numbers in Atlantic (*Salmo salar*) and Chinook (*Oncorhynchus tshawytscha*) salmon compared to other salmonids (12,700 and 13,650, respectively).

Freshwater species of the same genera showed much lower numbers of lincRNAs, which could be interpreted as evidence that anadromous species may possess adaptations associated with life history, as has been suggested for salmonids coding genes with higher diversification rates [18]. Although tantalising, this possibility would require ecological analyses, life-history comparisons, or phylogenetic analyses. Differences in genome annotation quality provide an equally plausible explanation and cannot currently be excluded [12,30].

LincRNAs were enriched near chromosome ends and less abundant around centromeric regions (Figure 1B). Similar patterns have been reported in other vertebrates [31], although the mechanisms generating this distribution remain poorly understood. Further analyses incorporating recombination-rate and transposable-element datasets will be necessary to determine the causes of this pattern in salmonids.

Sequence similarity among putative lincRNA orthologues and ohnologues declined markedly with increasing phylogenetic distance, paralleling the general pattern observed for coding genes but occurring at a substantially faster rate. This trend is consistent with the predominance of neutral sequence drift and the generally weaker evolutionary constraints acting on lincRNA sequences compared with coding regions [32]. Accordingly, putative orthologues shared across all salmonid species were far less common than the orthologues identified among protein-coding genes. Depending on the species comparison, only 11–68 lincRNAs showed detectable similarity across all salmonids and the outgroup northern pike, whereas a few hundred conserved loci were identified among salmonids and progressively higher numbers within individual genera (Figure 2A). The sharp decline in detectable orthology across deeper evolutionary timescales suggests extensive sequence divergence following lineage separation. Many putative lincRNA homologues diverged beyond the 70% similarity threshold used in this study. This was observed even among lineages that radiated after the salmonid-specific WGD. This observation is consistent with previous studies showing that lincRNA loci are typically subject to weaker purifying selection than coding genes and consequently accumulate substitutions, insertions, and deletions more rapidly over evolutionary time [33,34]. Indeed, lincRNAs are well known to exhibit lower sequence conservation and higher turnover rates than coding genes across vertebrate genomes, with functional conservation suggested to be maintained through preserved genomic context, promoter architecture, expression profiles, or structural features rather than extensive primary sequence similarity [33,34].

Despite limited sequence conservation, a subset of similar lincRNAs persists across distant salmonid lineages. This retention suggests that some loci may be under selective pressure. In these cases, conservation may reside in genomic position, RNA structure or regulatory interactions rather than primary sequence [35]. While synteny often reflects cis-regulatory roles near neighbouring genes [36,37], lincRNA synteny conservation is frequently lost in some species [38], and the percentage of lincRNA syntenic conservation is quite variable with evolutionary distances and evolutionary history. For instance, when comparisons were made between humans and mice (the most heavily studied), roughly 20–35% of human lincRNAs have a clear positionally conserved counterpart in the mouse genome, while when comparing zebrafish with humans, some stringent syntenic analyses reduce this to just 1 to 2% or up to 25% when the criteria are relaxed [37,39]. Synteny conservation values for putative orthologues fall within this range, while putative ohnologues are slightly higher, as expected for closely related species. Some authors have suggested that transposable element enrichment in lincRNA loci may accelerate recombination and synteny disruption [33,40]. However, we have to take into consideration that in the present work we only assessed the synteny of annotated genes surrounding lincRNAs, omitting microsynteny, which some authors have suggested is more conserved [41]. The preservation of both secondary structure and syntenic position identifies these loci as promising candidates for functional conservation. However, direct evidence for conserved biological functions will require further testing [42]. The lower retention estimates may partly result from the conservative BLAST threshold used. Divergent lincRNA ohnologues may fall below 70% sequence similarity and therefore escape detection. However, previous tests using a lower similarity threshold in non-salmonid data from our laboratory indicated a dramatic increase in BLAST false positives. The synteny analyses provide additional support for a subset of candidate lincRNA ohnologues identified from chromosomal location and sequence similarity. While chromosomal position alone cannot distinguish between all duplication mechanisms, the recovery of conserved syntenic relationships in a proportion of candidate duplicates is consistent with their origin from large-scale duplication events. Furthermore, candidates simultaneously supported by sequence similarity, conserved synteny and structural conservation represent particularly promising targets for future functional validation. Nevertheless, definitive assignment of WGD origin would require integration of chromosome homology information, phylogenetic reconstruction and/or synonymous divergence analyses.

Secondary structure conservation showed a moderate association with sequence similarity (ρ = −0.45; *p* = 2.2 × 10^−16^), with conserved structures being more likely between pairs when sequence similarity approached or exceeded ~80%, indicating that sequence similarity contributes to, but does not fully explain, structural conservation (Figure 3A,B). This pattern is consistent with the sequence-dependence of RNA folding and agrees with previous studies showing that lncRNA structural conservation generally decreases as sequence divergence accumulates [14]. Accordingly, ohnologues exhibited higher levels of secondary-structure conservation than orthologues (Figure 4A), likely reflecting their overall greater sequence similarity resulting from their more recent common ancestry following SWGD. The exception observed in Chinook salmon, with two distinct groups displaying different levels of structural conservation among ohnologues, suggests that additional lineage-specific factors contribute to the structural evolution of duplicated lincRNAs. Nevertheless, the broad concordance between sequence and structural conservation supports the view that sequence divergence is a major determinant of lincRNA structural evolution. The coexistence of highly conserved and poorly conserved ohnologues suggests heterogeneous evolutionary trajectories following WGD [43].

Approximately 50% of the ohnologue pairs with highly conserved secondary structures also retained local synteny (Figure 4D), suggesting a partial link between structural conservation and the maintenance of genomic context, which supports the hypothesis that these candidate ohnologues with conserved synteny and secondary structures are likely derived from the SWGD ohnologues. Although this association does not demonstrate functional conservation, it is noteworthy given that many lincRNAs are known to regulate neighbouring genes in cis- through sequence-, transcription-, or locus-dependent mechanisms [44]. If the function of these lincRNAs is mediated by RNA structure [45], the preservation of both secondary structure and syntenic position may also indicate the retention of regulatory roles. Under this scenario, conservation of the surrounding genomic environment could reflect the continued association of these lincRNAs with neighbouring target genes. However, functional studies will be required to determine whether the structurally conserved and syntenic lincRNAs identified here indeed retain conserved regulatory functions.

The correlation analysis developed in the present study, while limited to Atlantic salmon and rainbow trout, provides insights into the potential mechanisms underlying lincRNA retention after duplication events such as WGD. The majority of transcriptomic comparisons showed very weak correlation (between −0.1 and 0.1 Pearson correlation values; Figure 5A) compared with coding genes, where a much higher number of positive correlations (*ρ* > 0.5) were found. The generally weak transcriptional correlations among lincRNA ohnologues are consistent with subfunctionalisation or neofunctionalisation [43], suggesting that duplicated lincRNAs may have diverged more rapidly than duplicated coding genes. However, the limited amount of transcriptomic data (only two species), the generally low expression of lincRNAs, and the lack of transcriptomic data for northern pike (pre-duplicated outgroup) prevent robust conclusions regarding the mechanisms responsible for lincRNA retention. Caution is warranted when extrapolating these patterns across salmonids because equivalent transcriptomic datasets were unavailable for the remaining species.

The lincRNA–miRNA interaction analysis also showed an interesting perspective on lincRNA conservation. Our results indicated that lincRNA orthologues conserved at the sequence level between the five salmonid species investigated also share similar potential miRNA interactions. That they share similar miRNA interactions is not completely unexpected, as the detection of conserved lincRNAs was based on sequence similarity; therefore, it is quite likely that regions conserved across the species also contain miRNA interactions. Despite the lack of miRNA transcriptomic data and further experimental validation, the results suggest that a small subset of lincRNAs conserved across species may possess a potential sponge function [46]. Although lincRNA conservation across species is generally low, it has also been observed that brain lincRNAs are among those better conserved, including some lincRNA sponges such as HOXA-AS3 (a sponge of *miR-455*) [47]. The present work showed that putative lincRNA orthologues conserved across salmonids (excluding northern pike) had a conserved potential interaction with 18 miRNAs, with many of them having dual roles in brain and bone development, such as *miR-455*, *miR-365*, *miR-124*, or *miR-153*. *miR-455* (a miRNA showing a high proportion of strong interactions) has been linked to neuroprotection, synapse preservation and even spatial recognition [48,49], while in relation to bone, it helps guide early embryonic limb and cartilage development; its expression is often altered in adult osteoarthritis, indicating its response to joint stress [50]. *miR-153* has been associated with Alzheimer’s and Parkinson’s disease pathogenesis [51] and has also been implicated in memory processes and neurogenesis [52,53]. In human bone, *miR-153* targets *runx2* and *bmpr2* to regulate osteoblast differentiation [54,55]. *miR-365a* in humans increases rapidly in chondrocytes in response to mechanical loading [56], whereas in the brain it has been reported to inhibit astrocyte-to-neuron conversion through targeting *pax6* [57]. *miR-124* is one of the best-studied miRNAs in mammalian brain function, playing an important role in neural identity and maturation [58,59]. The conservation of lincRNAs with strong interactions towards brain- and bone-related miRNAs could be explained by at least two non-exclusive scenarios. First, because the present study focused on lincRNAs shared among salmonids and likely derived from the salmonid-specific WGD, conserved brain-related lincRNAs may simply represent a subset of transcripts evolving under stronger functional constraints, resulting in lower sequence divergence and consequently greater conservation of predicted miRNA interactions. Second, salmonids possess several distinctive neural and skeletal characteristics compared with many other teleost fishes [60,61,62,63]. Although the present study does not directly test links between these traits and lincRNA evolution, some developmental regulatory networks may have experienced greater evolutionary conservation. Future comparative analyses including additional teleost groups will be necessary to determine whether brain- and bone-related lincRNA networks are indeed preferentially conserved in salmonids. Salmonids display distinctive neural and skeletal features compared with many teleost species, including specialised migratory behaviours and the presence of acellular bone [64,65]. Whether these biological characteristics have contributed to the long-term conservation of specific lincRNA-mediated regulatory networks remains unknown. Nevertheless, the enrichment of conserved interactions involving miRNAs associated with neural and skeletal development suggests that these biological processes represent promising targets for future functional investigations.

While not as abundant as interactions involving bone- and brain-related miRNAs, the present work also identified multiple predicted interactions with miRNAs commonly associated with tumour suppression, including *let7*, *miR-124*, *miR-98*, and *miR-15a*. Previous studies have suggested that dosage-sensitive genes, including tumour suppressors, may be preferentially retained after WGD [66]. Under this framework, the conservation of some lincRNA–miRNA interactions may reflect the maintenance of regulatory networks involved in post-WGD dosage balance. Similarly, miRNAs have been proposed to contribute to the regulation of duplicated genes during rediploidization by buffering expression differences between duplicated copies [67]. Although the present data do not directly test these hypotheses, the observed conservation of several tumour-suppressor-associated miRNA interactions is consistent with a possible role in the stabilisation of post-WGD regulatory networks.

In addition to brain-, bone-, and tumour-suppression-related miRNAs, the strong predicted interactions with the muscle-related miRNA *miR-133a* were also relatively abundant. *miR-133a* is a conserved myomiR with key roles in skeletal and cardiac muscle [68,69]. Although salmonids possess physiological and morphological adaptations that support sustained swimming performance and high cardiac output [70,71,72], the present data do not allow direct links between these traits and lincRNA conservation to be established. Nevertheless, the current identification of *miR-133a* among the strongest predicted interactions suggests that muscle-related regulatory functions represent another potentially conserved aspect of salmonid lincRNA biology.

It is worth noting that there was little conservation of direct interactions between salmonid potential lincRNAs and bone and brain mRNAs. Only *col1a2* and *spp1* showed significant interaction with lincRNAs across species, with *col1a2* and *spp1* being involved in bone matrix [52] and brain development, respectively [73]. This low conservation of lincRNA–mRNA interactions may be a consequence of the rapid evolution of lincRNA sequences, which would be fast enough to affect mRNA interactions, where larger regions of sequence complementarity are required than for miRNA interactions, thereby facilitating the conservation of miRNA-sponge functions.

Despite the lack of experimental validation supporting previous findings, data generated during the AQUAFAANG initiative provided indirect support for the potential involvement of some conserved lincRNAs in brain and bone-related processes in salmonids. The AQUAFAANG embryonic development and tissue expression datasets for Atlantic salmon and rainbow trout showed that some conserved salmonid lincRNAs had comparatively higher expression in brain tissue and were also more transcribed during embryonic stages associated with nervous system and skeletal development. Rainbow trout exhibited a similar, albeit less clear, pattern. While these observations do not demonstrate a functional role, they are consistent with the hypothesis that at least a subset of the conserved lincRNAs may participate in bone and brain function and development [74].

Additional support comes from the strong positive correlations (*ρ* > 0.8) observed between the expression of conserved lincRNAs exhibiting strong miRNA interactions and key genes involved in brain and bone development in both Atlantic salmon and rainbow trout. The absence of similarly strong negative correlations (*ρ* < −0.8) is compatible with a model in which some lincRNAs act as competing endogenous RNAs or “miRNA sponges”, reducing miRNA availability and thereby favouring the accumulation of the target transcripts. However, correlation alone cannot distinguish between direct and indirect regulatory relationships, and the lack of matching miRNA expression data prevents a comprehensive reconstruction of the underlying regulatory networks. Consequently, the proposed sponge activity should be regarded as a working hypothesis requiring experimental validation [45]. Future studies should experimentally validate candidate lincRNA functions through gene disruption or knockdown approaches (among others) to verify putative miRNA-sponge activity, together with integrated miRNA–mRNA–lincRNA transcriptomic profiling.

## 4. Materials and Methods

### 4.1. Retrieval of lincRNAs and Detection of Highly Similar lincRNAs

All annotated lincRNA, mRNA, and miRNA FASTA sequences (Supplementary File S8) and chromosomal positions (Supplementary File S9) were retrieved from five salmonid genomes available in BioMart Ensembl 114 (https://may2025.archive.ensembl.org/index.html; accessed on 15 July 2025), including rainbow trout (*Oncorhynchus mykiss*), brown trout (*Salmo trutta*), Chinook salmon (*Oncorhynchus tshawytscha*), coho salmon (*Oncorhynchus kisutch*), and Atlantic salmon (*Salmo salar*), as well as their closest non-duplicated relative, the northern pike (*Esox lucius*). For comparative purposes, sequences of 340 protein-coding genes (including duplicates derived from TWGD, SWGD, and orthologues) were randomly retrieved (Supplementary File S10).

All within- and between-species sequence BLAST searches were performed to detect highly similar lincRNAs using the local BLASTn implementation in BioEdit v7.2.5 [75]. All BLASTn searches were performed with an e-value threshold of <1 × 10^−30^ (BioEdit and BLAST+ default parameters). The BLASTn results were used to identify syntenic blocks between and within species (methods Section 4.2). LincRNA pairs from BLASTn were further filtered to detect highly conserved lincRNAs likely originating from duplication events. Consequently, a conservative threshold of 70% similarity between pairs and a minimum alignment length of 100 nucleotides (to reduce the number of alignments in short repetitive regions) were used. The percentage of identity and minimal alignment were chosen based on previous in-house tests on non-salmonid datasets generated by our group. Those previous tests showed that lower identity cut-offs (<70%) substantially increased the number of matches but negatively affected other downstream analyses such as secondary structure conservation between pairs. To minimise false-positive matches, only reciprocal best hits satisfying all similarity criteria were retained. In addition, the use of a stringent BLAST e-value threshold (<1 × 10^−30^) already estimates the expected number of matches occurring by chance within the searched database, and no additional multiple-testing correction was applied for the homology-identification step.

### 4.2. Duplicated Classification and Synteny Conservation

The selected highly similar lincRNA duplicates within each species were classified based on their genomic location within the salmonid genome using physical coordinates obtained from the ensembl.org BioMart platform. Duplicates located on the same chromosome were considered putative local duplicates (LDs). Duplicates located on different chromosomes were considered putative WGD-derived ohnologues. Candidate lincRNA ohnologues were initially defined from sequence similarity and chromosomal location. Conserved synteny was then assessed using MCScanX (v1.0.0) (default parameters) [76] and manual inspection of the surrounding 1 Mb genomic regions in the Ensembl genome browser (accessed on 15 July 2025) as an independent criterion for evaluating the inferred duplication relationships.

### 4.3. Transcriptomic Analysis

All Atlantic salmon and rainbow trout transcriptomic data were generated by the Dr Macqueen Research Team at the Roslin Institute, University of Edinburgh, as part of the EU-funded AQUA-FAANG project. Transcriptomes were obtained from 11 different embryonic stages (early blastula, mid-blastula, late blastula, early gastrulation, mid-gastrulation, late gastrulation, early somatogenesis, mid-somatogenesis, late somatogenesis, early eye, and late eye formation) from three pools of embryos for each stage (*n* = 3 per stage) and 6 adult tissues (fast skeletal muscle, distal intestine, liver, head kidney, gill, and brain) from 12 animals (*n* = 12 per tissue). Details of RNA extraction and sequencing can be found in [29], and expression data can be found in SalmoBase (https://salmobase.org/; accessed on 1 March 2025) However, expression data for equivalent developmental stages and tissues were only available for Atlantic salmon and rainbow trout, preventing transcriptomic analyses from being extended to all species investigated in the present study.

### 4.4. Estimation of Structural Similarity Among lincRNA Pairs and lincRNA–mRNA–miRNA Sequence Interactions

Secondary structure conservation between lincRNA pairs (orthologues and ohnologues) was assessed using the CROSSalign web server (tools.tartaglialab.com/, accessed on 1 March 2026) [14] with the standard Dynamic Time Warping (DTW) mode. Only highly similar lincRNA pairs with ≥50% sequence alignment were compared for secondary structure conservation, based on a preliminary test performed by our group in non-salmonid species, which showed that the percentage of successful secondary structure reconstruction decreased with lower alignment percentages; therefore, we used ≥50% sequence alignment to increase efficiency. Based on [14], two lincRNAs were considered structurally conserved when the CROSSalign comparison score was <0.09 and the associated *p* < 0.05. For descriptive purposes only, conserved pairs were further subdivided into low/weakly conserved (0.09 to 0.05), moderately conserved (0.05 to 0.03), and highly conserved (<0.03) categories. These additional categories were introduced in the present study to facilitate interpretation and do not represent established CROSSalign thresholds. Interactions between lincRNA and miRNA were estimated using miRanda from the Galaxy platform (v.3.3a) [77] with a minimal normalised interaction energy threshold of –50 kcal/mol. Interactions between lincRNAs and mRNAs at the sequence level were estimated using lncTar (v.1.0) software with a normalised interaction energy (ndG) threshold of <−0.10 kcal × mol^−1^ × nt^−1^ [78].

### 4.5. Statistical Analysis

Unless otherwise indicated, transcription levels were presented as mean normalised expression values in transcripts per million (TPM) and as mean values ± standard deviation (SD). Pearson correlations for transcriptomic data were calculated using the RStudio (2024.12.1 + 563) corr package (v.0.95) [79]. Average TPM values for each developmental stage and tissue were used for correlation analyses. Differences among density distributions were evaluated using two-sided Kolmogorov–Smirnov (K-S) tests. Differences in proportions of structurally conserved lincRNA pairs were evaluated using χ^2^ tests. For differences in lincRNA transcription among tissues, a one-way ANOVA followed by Tukey’s post hoc test was applied. In all cases, the significance level was set at 0.05. For Pearson correlation and ANOVA analyses, normality and homogeneity of variances were verified by using Shapiro–Wilk and Levene’s tests, respectively.

All graphs were generated using ggplot2 [80], and confidence intervals for regressions were generated using the default functions implemented in ggplot2 (v.3.5.1) options.

## 5. Conclusions

The present study reveals a heterogeneous landscape of lincRNAs across salmonid genomes, likely partially shaped by the salmonid-specific whole-genome duplication. LincRNAs exhibit low sequence conservation that decreases with phylogenetic distance, with only a limited number of possible lincRNA orthologues shared across all salmonid species. Conservation of local synteny was higher among putative ohnologues than among orthologues, and putative ohnologues also showed a higher proportion of conserved secondary structure.

Although duplicated lincRNAs are more abundant in salmonids than in *Esox lucius*, consistent with an effect of the salmonid-specific WGD, only a subset of candidate duplicates was independently supported by conserved syntenic relationships. This suggests that the identified lincRNA ohnologues should be considered strong candidates rather than definitive WGD-derived duplicates.

Structural conservation is restricted to highly similar sequences, and expression analyses reveal low transcription levels and weak correlations between duplicates, consistent with widespread regulatory divergence. However, conserved lincRNAs among salmonids appeared to show strong interactions with miRNAs involved in tumour suppression, brain, bone, and muscle development, simultaneously showing transcriptional patterns correlated with brain and bone function. These observations are consistent with the possibility that some lincRNAs associated with brain and bone-related regulatory pathways have been retained more frequently than others, although experimental validation will be necessary to determine whether this reflects stronger evolutionary constraints.

## Figures and Tables

**Table 1:** Percentage of conserved synteny between lincRNA orthologue pairs across species.

lincRNA Pairs Comparison	Synteny	No-Synteny
*Ots:Oki*	16	83
*Omy:Ots*	11	89
*Omy:Oki*	12	88
*Ssa:Stu*	16	84
*Ssa:Ots*	8	91
*Ssa:Oki*	12	87
*Ssa:Elu*	5	95

*Ots*: *Oncorhynchus tshawytscha*; *Oki*: *Oncorhynchus kisutch*; *Omy*: *Oncorhynchus mykiss*; *Ssa*: *Salmo salar*; *Stu*: *Salmo trutta*; *Elu*: *Esox lucius*.

**Table 2:** Percentage of conserved synteny between lincRNA ohnologue pairs across species.

	*Ssa*	*Stu*	*Omy*	*Oki*	*Ots*
Genes in synteny blocks ^1^	49.6%	62.6%	54.5%	59.45%	56.3%
lincRNA in synteny blocks ^2^	8.1%	32.9%	23.4%	21.1%	11.8%
Number of highly similar lincRNAs ^3^	920	559	857	931	1033
Highly similar lincRNA in synteny blocks ^4^	8.1%	15%	12.1%	10%	11%

*Ssa*: *Salmo salar*; *Stu*: *Salmo trutta*; *Omy*: *Oncorhynchus mykiss*; *Oki*: *Oncorhynchus kisutch*; *Ots*: *Oncorhynchus tshawytscha*. ^1^ Percentage of all genes allocated in synteny blocks by MCScanX. ^2^ Percentage of all genomic lincRNAs allocated in synteny blocks (lincRNAs in synteny blocks/total lincRNAs in the genome). ^3^ Number of lincRNAs obtained from autoBLASTn analysis with e-value < 1 × 10^−30^, minimum similarity > 70%, and minimum alignment of >100 nucleotides. ^4^ Percentage of highly similar lincRNAs forming synteny blocks (conserved lincRNAs in synteny blocks/total conserved lincRNAs × 100). All data from MCScanX analysis can be found in Supplementary File S2.

**Table 3:** Best lincRNA–miRNA interactions for salmonid species.

Species	miRNA	lincRNA	
	ID	ID	MFE
*Ssa*	*miR-455*	ENSSSAG00000089172	−105.28
	*miR-365a*	ENSSSAG00000094512	−99.21
	*miR-153-2*	ENSSSAG00000105779	−99.90
	*miR-124*	ENSSSAG00000093740	−101.78
*Omy*	*miR-455*	ENSOMYG00000047025	−110.8
	*miR-365a*	ENSOMYG00000024861	−95.23
	*miR-153-2*	ENSOMYG00000002003	−102.6
	*miR-124*	ENSOMYG00000035300	−51.84
*Ots*	*miR-455*	ENSOTSG00005000067	−87.63
	*miR-365a*	ENSOTSG00005006727	−84.56
	*miR-153-2*	ENSOTSG00005017513	−91.55
	*miR-124*	ENSOTSG00005037223	−84.77
*Oki*	*miR-455*	ENSOKIG00005014297	−111.2
	*miR-365a*	ENSOKIG00005031555	−98.99
	*miR-153-2*	ENSOKIG00005027572	−108.9
	*miR-124*	ENSOKIG00005010426	−83.52
*Stu*	*miR-455*	ENSSTUG00000003285	−104.7
	*miR-365a*	ENSSTUG00000049242	−98.75
	*miR-153-2*	ENSSTUG00000039218	−117.6
	*miR-124*	ENSSTUG00000042406	−79.24

Best miRNA–lincRNA interactions for four of the miRNAs detected in the present study. *Ssa*: *Salmo salar*; *Stu*: *Salmo trutta*; *Omy*: *Oncorhynchus mykiss*; *Oki*: *Oncorhynchus kisutch*; *Ots*: *Oncorhynchus tshawytscha*. MFE: minimal energy interaction between miRNA and lincRNA in kcal/mol.

**Table 4:** Best interactions between key bone and brain genes, miRNAs and lincRNAs.

Gene	Species	miRNA		lincRNA	
		ID	MFE	ID	ndG
*alpl*	*Ssa*	*miR-455*	−92.63	ENSSSAG00000004223	−0.10
	*Omy*	*miR-455*	−92.13	ENSOMYG00000027581	−0.12
	*Oki*	*miR-455*	−93.96	na	na
	*Ots*	*miR-455*	−92.13	na	na
	*Stu*	*miR-365a*	−96.27	na	na
*col1a2*	*Ssa*	*miR-455*	−94.91	ENSSSAG00000046988	−0.13
	*Omy*	*miR-455*	−94.68	ENSOMYG00000009373	−0.17
	*Oki*	*miR-455*	−98.2	ENSOKIG00005016895	−0.16
	*Ots*	*miR-455*	−97.18	na	na
	*Stu*	*miR-153a*	−99.31	ENSSTUG00000043917	−0.16
*hdac8*	*Ssa*	*miR-455*	−103.26	na	na
	*Omy*	*miR-455*	−96.02	ENSOMYG00000027430	−0.22
	*Oki*	*miR-455*	−95.05	na	na
	*Ots*	*miR-455*	−95.95	na	na
	*Stu*	*miR-455*	−103.26	ENSSTUG00000002071	−0.11
*sparc*	*Ssa*	*miR-365a*	−81.63	na	na
	*Omy*	*miR-455*	−98.65	ENSOMYG00000036119	−0.11
	*Oki*	*miR-365a*	−83.79	na	na
	*Ots*	*miR-365a*	−83.79	na	na
	*Stu*	*miR-140*	−85.55	na	na
*spp1*	*Ssa*	*miR-9-3*	−97.49	ENSSSAG00000100947	−0.60
	*Omy*	*miR-153-2*	−89.93	ENSOMYG00000046677	−0.47
	*Oki*	*miR-153-2*	−91.26	ENSOKIG00005014310	−0.16
	*Ots*	*miR-153-2*	−89.93	ENSOTSG00005011947	−0.24
	*Stu*	*miR-455*	−94.4	ENSSTUG00000036943	−0.13

*Ssa*: *Salmo salar*; *Stu*: *Salmo trutta*; *Omy*: *Oncorhynchus mykiss*; *Oki*: *Oncorhynchus kisutch*; *Ots*: *Oncorhynchus tshawytscha*. ndG: energy interaction between mRNA and lincRNA normalised by the length of the lincRNA (kcal × mol^−1^ × nt^−1^). MFE: minimal energy interaction between miRNA and mRNA (kcal/mol).

## Data Availability

The original contributions presented in this study are included in the article/Supplementary Materials. Further inquiries can be directed to the corresponding author.

## Supplementary Materials

The following supporting information can be downloaded at: https://www.mdpi.com/article/10.3390/ijms27177861/s1.
