# Supplementary material for: Exploring the Effect of Whole-Genome Duplication on Salmonid LincRNA Repertoire
Source: Int J Mol Sci. 2026 Sep 2;27(17):7861. doi: 10.3390/ijms27177861 (PMC13566215; doi:10.3390/ijms27177861)
Supplement: Supplementary file 1 [file ijms-27-07861-s001.zip › Supplementary_File S3.pdf]

Supplementary File S3. Statistics Figure S3, 4 and 5.

Figure S3.

	Corr	pval
All	-0.45	$2.2e^{-16}$
Ohnologues	-0.62	$1.02e^{-45}$
Orthologues	-0.41	$1.99e^{-14}$
LD	-0.31	$4.8e^{-4}$

Figure S4.

A.

KS-test	pval
Ohno:Ortho	$6.4e^{-10}$
Ohno:LD	$2.2e^{-16}$
Ortho:LD	$4.8e^{-5}$

B.

KS-test	pval
Onco/salmo	0.022
Onco_vs_salmo/Salmo	0.019
Onco_vs_salmo/Onco	0.018

C.

KS-test	pval
Oki:Omy	$8.8e^{-03}$
Oki:Ots	$1.6e^{-04}$
Oki:Ssa	$7.7e\text{-}^{02}$
Oki:Stu	$4.4e^{-01}$
Omy:Ots	$1.5e\text{-}^{10}$
Omy:Ssa	$2.1e^{-01}$
Omy:Stu	$1.8e^{-01}$
Ots:Ssa	$3.5e^{-06}$
Ots:Stu	$3.5e^{-05}$
Ssa:Stu	$6.6e^{-01}$

D.

Chi-square	pval
High	$6.1e\text{-}^{4}$
Medium	0.008
Low	0.045

Figure S5.

A.

KS-test	pval
Ortho:Ohno_Omy	$2.6e^{-11}$
Ortho:Ohno_Ssa	$2.2e^{-16}$
Ohno_Omy:Ohno_Ssa	$8.2e^{-5}$

B.

KS-test	pval
Ortho:Ohno_Omy	$7.9e^{-08}$
Ortho:Ohno_Ssa	0.06
Ohno_Omy:Ohno_Ssa	0.0005

C.

KS-test	pval
Ortho:Ohno_Omy	0.02
Ortho:Ohno_Ssa	$9.4e^{-7}$
Ohno_Omy:Ohno_Ssa	$5.1e^{-6}$

D.

KS-test	pval
Ortho:Ohno_Omy	0.03
Ortho:Ohno_Ssa	0.05
Ohno_Omy:Ohno_Ssa	0.42
