# Supplementary material for: Exploring the Effect of Whole-Genome Duplication on Salmonid LincRNA Repertoire
Source: Int J Mol Sci. 2026 Sep 2;27(17):7861. doi: 10.3390/ijms27177861 (PMC13566215; doi:10.3390/ijms27177861)
Supplement: Supplementary file 1 [file ijms-27-07861-s001.zip › Supplementary_File_S4.zip!/Supplementary_File_S4/Supplementary file S4c_density_distribution_TPM.pdf]

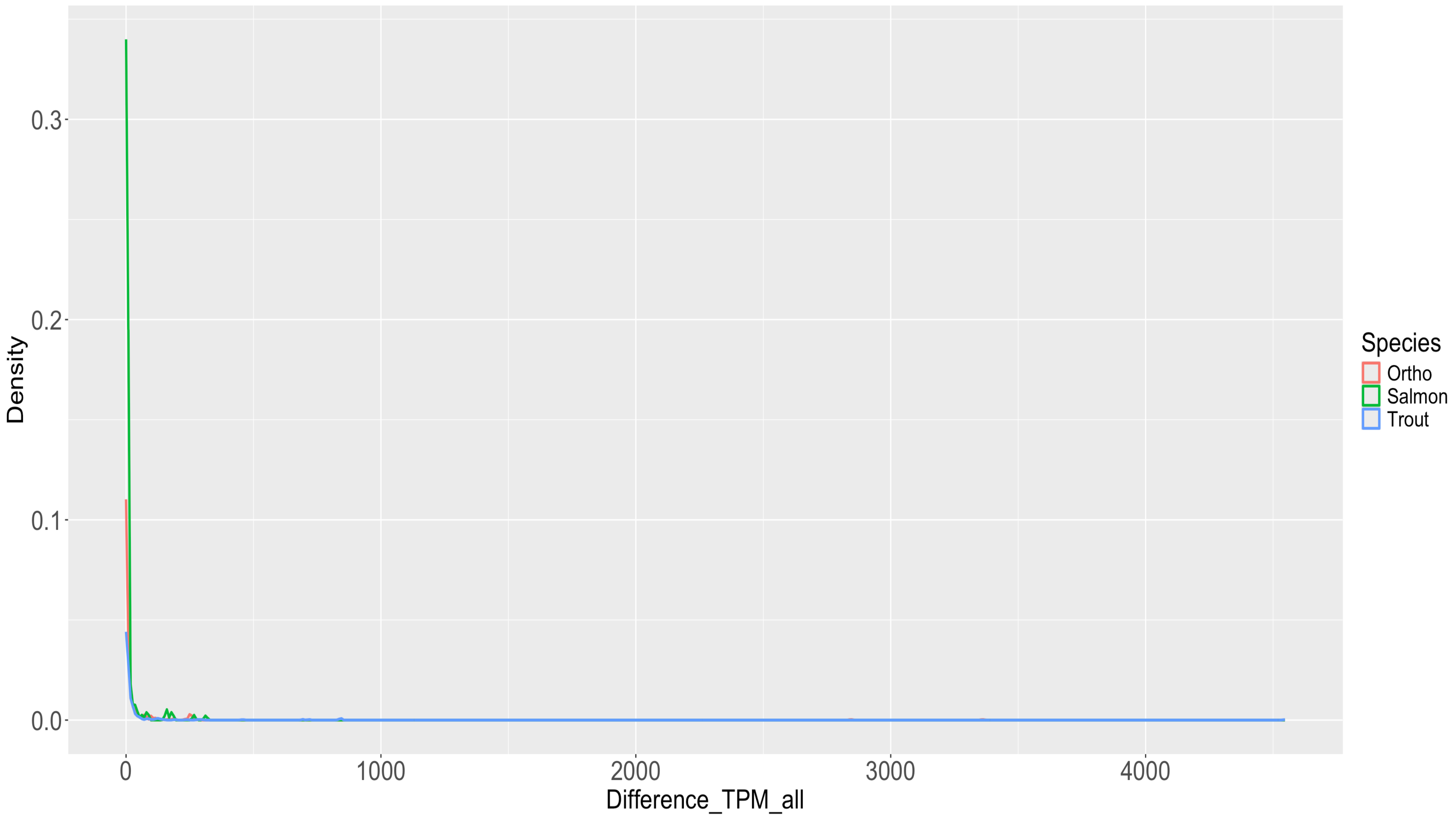

Density
0.3
0.2
0.1
0.0
0
1000
2000
3000
4000
Difference_TPM_all
Species
Ortho
Salmon
Trout

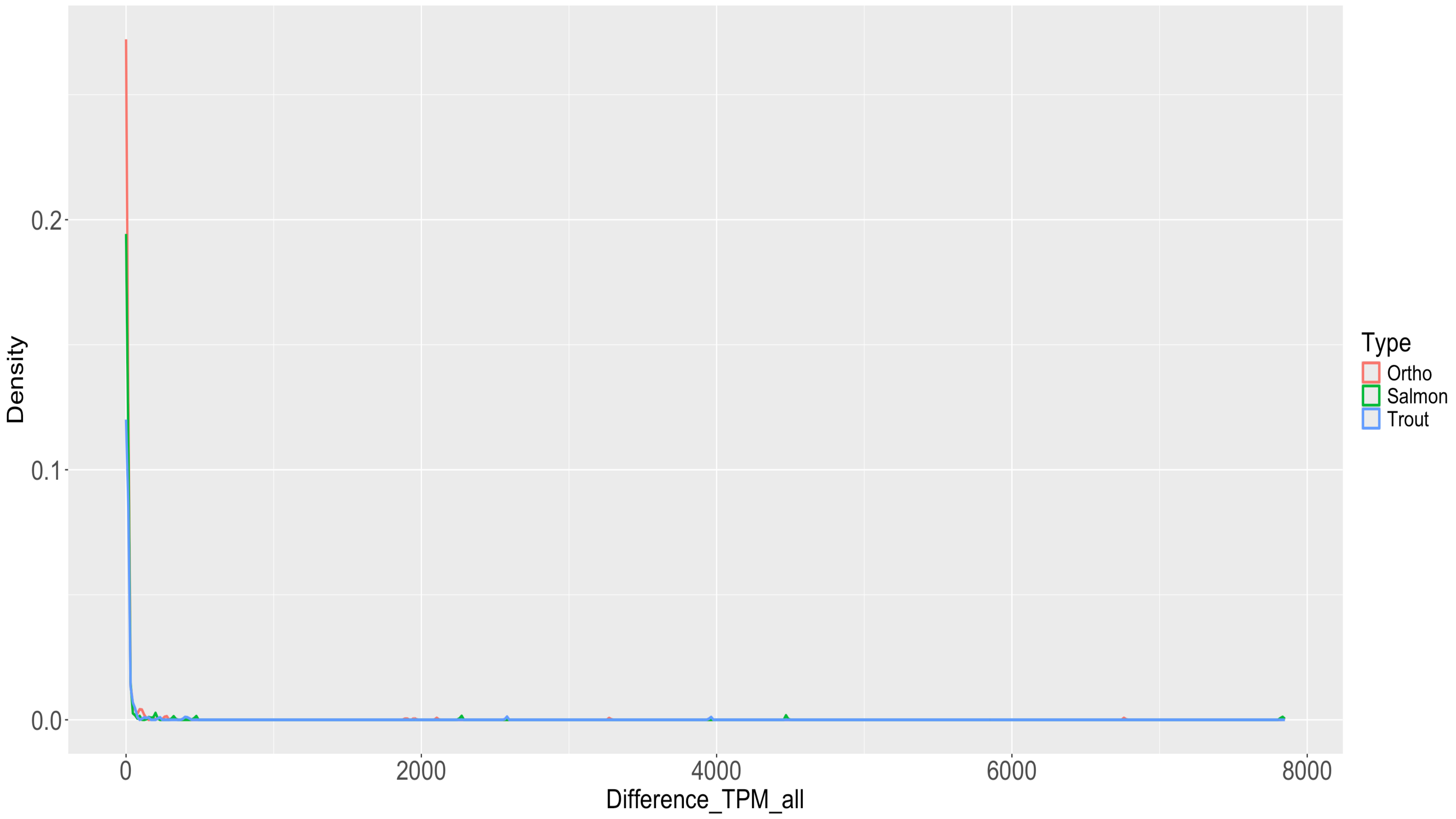

Density
0.2
0.1
0.0
0
2000
4000
6000
8000
Difference_TPM_all
Type
Ortho
Salmon
Trout
